# Observation of Néel-type skyrmions in acentric self-intercalated Cr_1+δ_Te_2_

**DOI:** 10.1038/s41467-022-31319-y

**Published:** 2022-07-08

**Authors:** Rana Saha, Holger L. Meyerheim, Börge Göbel, Binoy Krishna Hazra, Hakan Deniz, Katayoon Mohseni, Victor Antonov, Arthur Ernst, Dmitry Knyazev, Amilcar Bedoya-Pinto, Ingrid Mertig, Stuart S. P. Parkin

**Affiliations:** 1 Max Planck Institute of Microstructure Physics, Weinberg 2, Halle (Saale), 06120 Germany; 2 Institute of Physics, Martin Luther University, Halle-Wittenberg, Halle (Saale), 06120 Germany; 3 G. V. Kurdyumov Institute for Metal Physics, National Academy of Science of Ukraine, 03142 Kiev, Ukraine; 4 Institute for Theoretical Physics, Johannes Kepler University Linz, Altenberger Strasse 69, A-4040 Linz, Austria; 5 Instituto de Ciencia Molecular, Universidad de Valencia, Catedrático José Beltrán 2, Paterna, 46980 Spain

**Keywords:** Spintronics, Spintronics

## Abstract

Transition-metal dichalcogenides intercalated with 3*d*-transition metals within the van der Waals (vdW) gaps have been the focus of intense investigations owing to their fascinating structural and magnetic properties. At certain concentrations the intercalated atoms form ordered superstructures that exhibit ferromagnetic or anti-ferromagnetic ordering. Here we show that the self-intercalated compound Cr_1+δ_Te_2_ with δ ≈ 0.3 exhibits a new, so far unseen, three-dimensionally ordered (2×2×2) superstructure. Furthermore, high resolution X-ray diffraction reveals that there is an asymmetric occupation of the two inequivalent vdW gaps in the unit cell. The structure thus lacks inversion symmetry, which, thereby, allows for chiral non-collinear magnetic nanostructures. Indeed, Néel-type skyrmions are directly observed using Lorentz transmission electron microscopy. The skyrmions are stable within the accessible temperature range (100–200 K) as well as in zero magnetic field. The diameter of the Néel skyrmions increases with lamella thickness and varies with applied magnetic field, indicating the role of long-range dipole fields. Our studies show that self-intercalation in vdW materials is a novel route to the formation of synthetic non-collinear spin textures.

## Abstract (web summary)

Here, Saha et al. show that self-intercalation of e_2_Cr atoms in CrTe_2_ create an asymmetry in the number of atoms intercalated in the van der Waals gaps between the layers of CrTe_2_. This inversion symmetry breaking leads to non-collinear spin-textures and Néel-type magnetic skyrmions over a wide temperature range.

## Introduction

Within the group of magnetic and non-magnetic van der Waals (vdW) materials^1,2^, transition metal dichalcogenides (TMDs) represent a unique class of materials due to their layered arrangements and structural flexibility, the latter related to their ability to intercalate atoms into the vdW gaps^3–6^. Although vdW materials and TMDs have been studied for some time, more recently they have attracted intense interest owing to their remarkable magnetic properties in atomically thin layers^7–10^. Of the large number of TMDs those which have been reported to exhibit magnetic long-range order include MnSe_x_^11^ and CrTe_2_^12^. For the latter, a considerable number of studies concerning the thickness-dependent magnetic properties have been carried out, albeit with a notable scatter in the results reported with regard to the thickness dependence of the Curie temperature, the easy axis of magnetization, as well as the concentration of intercalants^13–18^. Most importantly, in all these previous reports the crystal structures in all thickness regimes were assumed to be highly symmetric belonging to the space group (SGR) *P*$\bar{3}$*m*, which is inversion symmetric. Inversion symmetry rules out many important physical properties which are related to polar tensors of odd rank and axial tensors of even rank^19^. In particular, the latter is related to the stabilization of non-collinear magnetism where the Dzyaloshinskii-Moriya interaction (DMI)^20–22^ plays a decisive role. The DMI competes with the Heisenberg exchange interaction to stabilize various types of chiral magnetic textures in non-centrosymmetric magnetic materials^23–27^. Only very recently has it been observed that massive structural relaxations in TMDs are present in the two-dimensional (2D) limit. For example, in a single TaSe_2_ monolayer, which is otherwise non-magnetic, a reduction in symmetry gives rise to the appearance of chiral magnetic states^28^.

Here we show that in a bulk Cr_1+δ_Te_2_ (δ ≈ 0.3) single crystal the prior assumption of an inversion symmetric structure is not correct and, as a consequence, a non-collinear chiral magnetic texture in the form of Néel-type skyrmions appears. While in the past only two-dimensional superstructures of type (2×2) and (√3×√3) were reported for ordered intercalated structures, here we observe a new, so far unknown three-dimensional (2×2×2) superstructure. It is characterized by the existence of two inequivalent vdW gaps, both partially filled by Cr atoms. As a consequence, the neighboring Te-Cr-Te triple layers (TLs) experience an asymmetric environment involving relaxations of the atomic positions along the vertical *c*-axis, thereby reducing the crystal symmetry from *P*$\bar{3}$*m* to *P*3*m*1, the latter being acentric. The latter has a *C*_3*v*_ point group that is compatible with the appearance of Néel-type magnetic textures, which we directly observe by Lorentz transmission electron microscopy (LTEM). Our results point to a new approach to stabilize non-collinear spin textures via self-intercalation in vdW magnets, which is distinct from schemes that make use of heteroepitaxial systems to induce skyrmions in vdW magnetic materials^29,30^.

## Results and discussion

Single crystals of Cr_1+δ_Te_2_ were grown by the flux zone growth method^31^. The structural and magnetic properties of the crystals were characterized by X-ray diffraction (XRD) and SQUID magnetometry, respectively. At room-temperature bulk CrTe_2_ was previously reported to crystallize in a trigonal structure with the centrosymmetric SGR $P\bar{3}m$^32^.

**Fig. 1:**
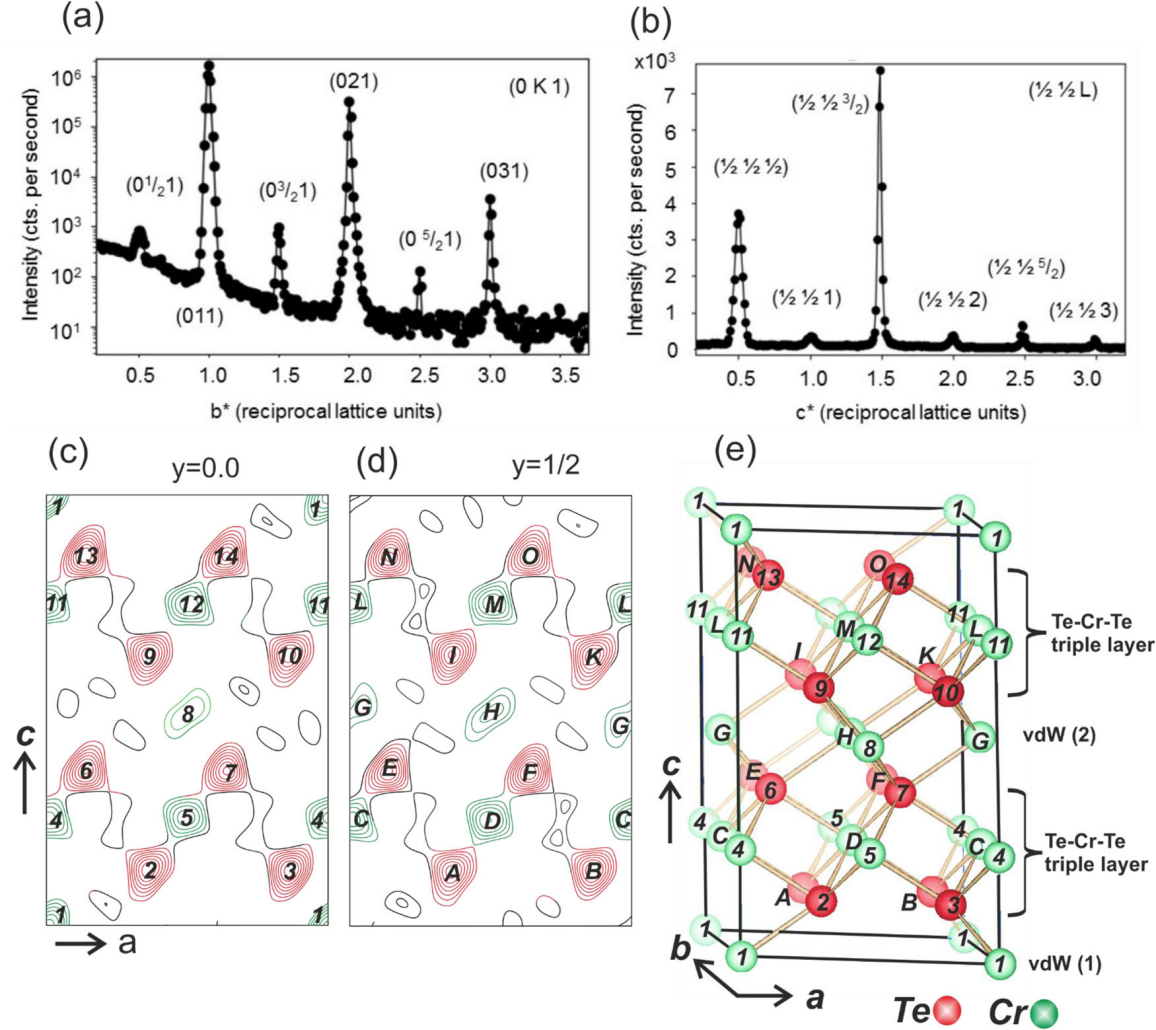
Structural characterization of Cr_1+δ_Te_2_ bulk crystal. **a** Line scan in reciprocal space along the b* axis at L = 1 crossing both integer and fractional order reflections of the (2×2×2) Cr_1+δ_Te_2_ bulk crystal. Reflections are labeled according to the (1×1×1) unit cell. The intensity (counts per second) is shown on a logarithmic scale. Note, that the half order reflections are weaker than the integer order ones by several orders of magnitude. **b** Line scan along superlattice reflections of type (½ ½ L) between L = 0.2 and 3.7 plotted on a linear scale. The observation of reflections with fractional indices along all three dimensions indicates the presence of a (2×2×2) superstructure. **c**–**e** Charge density contour map of the (2×2×2) Cr_1+δ_Te_2_ structure calculated for sections *y* = 0 (**c**) and *y* = 1/2 (**d**) within the *ac* plane compared with the structural model (**e**). Cr (green) and Te (red) atoms in section *y* = 0 and *y* = 1/2 are labeled by numbers and capital letters, respectively. The van der Waals (vdW) gaps (1) and (2) are not equivalent inducing vertical atomic shifts within the adjacent Te-Cr-Te triple layers, thereby reducing the symmetry from the centrosymmetric space group* P*$\bar{3}$*m* to the non-centrosymmetric space group *P*3*m*1.

In the following, we discuss XRD experiments carried out at room temperature, where a plate-like single crystal was investigated by collecting 158 symmetry independent reflections whose squared structure factor magnitudes (|F_obs_(HKL) | ^2^) span five orders of magnitude. In total, 36 reflections have integer HKL values, with respect to the trigonal unit cell whose lattice parameters (*a* = *b* = 3.91 Å, *c* = 5.99 Å) are close to those previously reported of *a* = 3.92 Å and *c* = 6.02 Å, respectively^32^. Figure 1a shows a line scan along the reciprocal b* axis at L = 1 reciprocal lattice units, i.e. reflections of type (0 K 1) for K varying between 0.2 and 3.7. We also observe reflections of type H = K = L = (2*n* + 1)/2, with *n* equal to an integer as shown in Fig. 1b, which, in combination with the three-fold symmetry of the diffraction pattern, is an unambiguous proof for the presence of a (2×2×2) superstructure [see also Supplementary Information (SI)]. The low intensity of the half order reflections arise from the fact that they originate from the difference between the Cr-intercalation structures in the two vdW gaps. At the half-order reflections there is an anti-phase relation between the waves scattered by the Cr atoms residing in the different vdW gaps. Therefore, they also show a strongly suppressed temperature dependence, as outlined in the Supplementary Information (SI). The half-order reflections are several orders of magnitude lower in intensity than the integer-order reflections.

In a least squares structural refinement which takes only the (1×1×1) integer order reflections into account the previously reported structural model is accurately reproduced in which the symmetry corresponds to the centrosymmetric SGR *P*$\bar{3}$*m* (SGR#164)^32,33^. Here, Te atoms are located at the Wyckoff site 2*d* at (*x*, *y*, *z*) = (1/2, 2/3, *z*) with *z *= 0.248 ± 0.001 and Cr atoms are located at the site 1*b* (0 0 ½). We obtain a good unweighted residuum (Ru)^34^ of 0.064 and a goodness of fit (GOF)^35^ near 1.0 provided that a fraction of 0.40 ± 0.10 atoms per unit cell of intercalated Cr is located in the vdW gap at the Wyckoff site 1*a* (0,0,0). On the basis of this structural model no significant improvement of the fit quality factors can be achieved by allowing for symmetry lowering such as e.g. by mapping the structure to the non-centrosymmetric SGR *P*3*m*1 (#156) and relaxing the *z*-positions of Te and Cr.

The presence of the half-order reflections along all three directions in reciprocal space directly indicates that the structure is considerably more complex. In Fig. 1c, d we show two sections (*y* = 0 and *y* = 1/2) of the charge density [*ρ*(*x*, *y*, *z*)] within the *ac* plane. The charge density is the Fourier-Transform (FT) of the observed structure factors [F_obs_(HKL)]. Only the preliminary structural model including two Te-Cr-Te triple layers (TLs) was considered as input for the calculation of the scattering phases. Thus, *ρ*(*x*, *y*, *z*) provides direct and quasi model-free evidence that the two vdW gaps (labeled by vdW(1) and vdW(2) in Fig. 1e) within the (2×2×2) unit cell are not equivalent. Charge densities (solid lines) in Fig. 1c, d are labeled by numbers for section (*y* = 0) and capital letters for section (*y* = ½). They relate to the correspondingly labeled atoms in the structural model shown in Fig. 1e. The most important result is that vdW(1) contains only one Cr atom at (0,0,0) (labeled by #1). By contrast, the intercalation in vdW(2) is characterized by three Cr sites, namely one position in section *y* = 0 at (½, 0, ½), labeled by #8 and two positions in section *y *= ½ labeled by (#G) and (#H) at (0, ½ ½) and (½ ½ ½), respectively.

All these intercalated Cr atoms directly appear in *ρ*(*x*, *y*, *z*). There are also several weaker peaks at asymmetric sites in *ρ*(*x*, *y*, *z*), which, however, are related to truncation errors. The positions of the atoms and their occupancy factors were refined in a least-squares fit which confirms the structural model shown in Fig. 1e, as outlined in the SI. The two symmetrically inequivalent TLs adjacent to the vdW gaps experience an asymmetric environment which induces vertical relaxations of the atoms along *z*. These relaxations are primarily responsible for lowering the symmetry from the centrosymmetric SGR $P\bar{3}m$ to the non-centrosymmetric SGR *P*3*m*1. We emphasize that this model corresponds to the average structure within one domain which we estimate is ~20 nm in size. On a length scale smaller than the domain size the symmetry of the structure is likely to be reduced due to the statistical occupancy of the different vdW sites and the presence of vacancies. The presence of two different Cr environments, namely Cr atoms bound to Te atoms within the TLs and Cr atoms located within the vdW gaps, is also revealed by X-ray absorption experiments, where two distinct features are observed at the Cr L_2,3_ absorption edge. In order to rule out multiplet splitting effects from a single Cr site^36^, we have carried out *ab*-initio calculations of the Cr absorption spectra with and without Cr occupation in the vdW gaps. These calculations support our supposition that additional Cr atoms occupy the vdW gap sites, as discussed in the SI (Figs. S6, S10 and text). Also, our calculations of the spin-resolved electronic structure reveal that the presence of intercalated Cr atoms contributes to the formation of non-collinear spin structures.

**Fig. 2:**
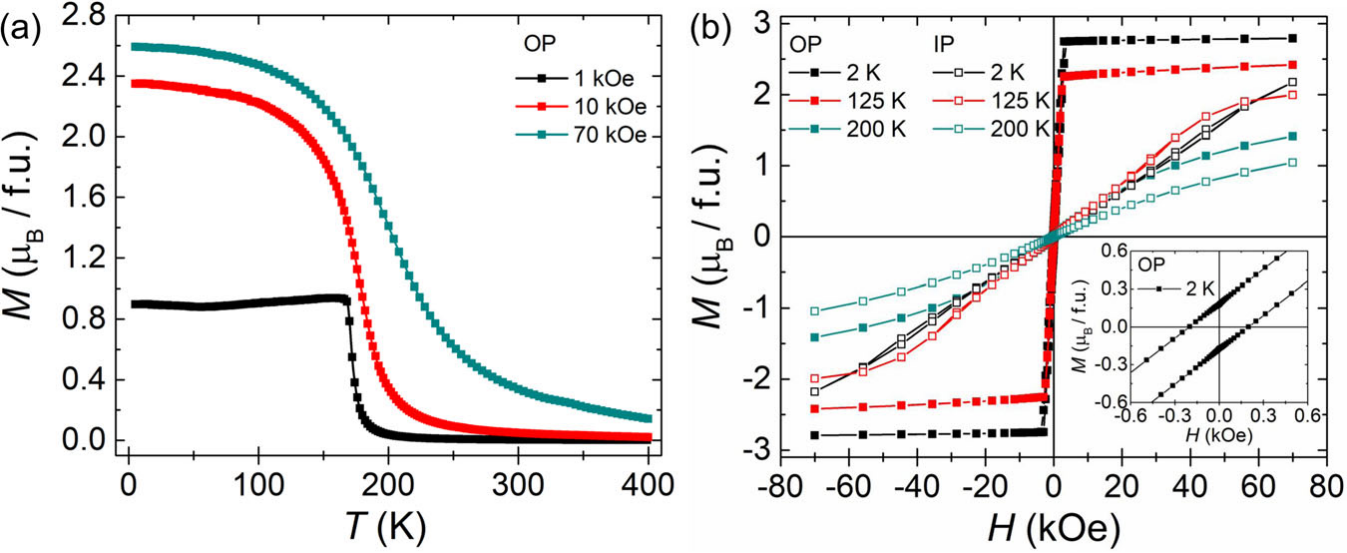
Magnetic characterization of a Cr_1+δ_Te_2_ bulk crystal. **a** Temperature (*T*) dependence of magnetization (*M*) of Cr_1+δ_Te_2_ for several applied magnetic fields (*H*) oriented along the out-of-plane direction [0001]. **b** Isothermal magnetization versus magnetic field oriented along in-plane (IP) and out-of-plane (OP) directions of the single crystalline platelet sample at 2, 125 and 200 K. Inset of b shows the magnified *M*(*H*) profile at 2 K for the OP direction.

Having assessed the presence of different Cr-sites, we proceed to characterize the magnetic properties of the crystal by magnetization measurements versus temperature and magnetic field, as shown in Fig. 2a, b. The temperature-dependent magnetization data, *M*(*T*), (Fig. 2a) were recorded after field-cooling the crystal from 400 to 2 K in the presence of a out-of-plane [0001] magnetic field of 1, 10 and 70 kOe, which indicates that the crystal magnetically orders below 200 K (see Fig. S11 in the SI for the in-plane *M*(*T*) data). Isothermal magnetization measurements as a function of magnetic field show a soft ferromagnetic hysteresis, as shown in Fig. 2b. From this figure we observe that at 2 K the *M*(*H*) data saturate at a lower magnetic field when the field was applied along [0001], whereas it does not saturate even if a magnetic field as large as 70 kOe is applied perpendicular to [0001]. The origin of such a large perpendicular anisotropy could be attributed to the presence of a high concentration of intercalated Cr atoms^17^ which is also supported by our calculations (see Figs. S8 and S9 and corresponding text in the SI). The lack of magnetic saturation when applying a large in-plane field has also been observed in element specific X-ray magnetic circular dichroism (XMCD) measurements at the Cr L_2,3_ edge of a freshly cleaved Cr_1+δ_Te_2_ crystal (Fig. S5 in SI). This indicates that the magnetic easy axis of the crystal is along the *c*-axis of the crystal. In the inset of Fig. 2b, a magnified region of the out of plane *M*(*H*) loop is shown, characterized by a finite coercive field of approximately 200 Oe at 2 K. In addition, temperature dependent XMCD measurements (Fig. S7 in SI) confirm the existence of a finite magnetic remanence (XMCD signal at *H* = 0 up to 200 K), indicating a long-range nature of the magnetic ordering.

**Fig. 3:**
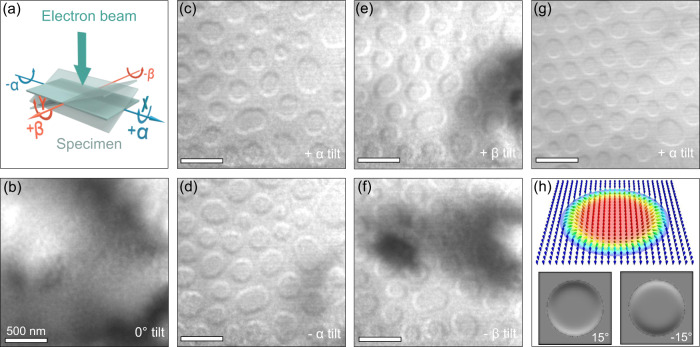
Néel-type magnetic textures in a thin lamella of Cr_1+δ_Te_2_ at 100 K. **a** Definition of tilt angles, *α* and *β,* that are defined with respect to the [0001] direction. When *α* = *β* = 0, the electron beam of the transmission electron microscope is transmitted along [0001]. **b** Lorentz transmission electron microscopy (LTEM) image under zero tilt. **c**–**f** LTEM images in zero magnetic field for (**c**–**d**) *α* = ±15° and (**e**–**f**) *β* = ±15°. All images are recorded at a defocus value of −1.5 mm. The large darkened areas correspond to bending contours. The lamella has a thickness of ≈ 82 nm. All micrographs are obtained from the same region. **g** LTEM image at a defocus value of −0.5 mm in zero magnetic field upon +4° tilting from the [01$\bar{1}$1] direction that corresponds to *α* = +33°, showing that the magnetic contrast is similar to that obtained at a higher defocus value. The lamella has a thickness of ≈ 75 nm. **h** Calculated LTEM contrast of the Néel skyrmion for a tilt of ±15° about the vertical axis in the image.

Since the inversion symmetry is broken in Cr_1+δ_Te_2_, a DMI is possible so that chiral non-collinear spin textures can form. In addition, magnetotransport measurements (see Fig. S12 in SI) reveal clear evidence for a topological Hall effect that is indicative of a non-trivial topological magnetic texture. In order to investigate the magnetic textures in Cr_1+δ_Te_2_ we carried out temperature- and field-dependent magnetic imaging experiments using LTEM. Specimens for LTEM studies were prepared in the form of thin [0001] oriented single crystalline Cr_1+δ_Te_2_ lamellae using focused ion-beam milling (FIB). The lamella was initially zero-field cooled from 368 to 100 K followed by LTEM imaging. Under this condition a cycloidal state was found as the ground state (see Fig. S13 in SI). With increasing magnetic field the cycloidal state gradually transforms into a field-polarized state without stabilizing any skyrmion state. A skyrmion state was stabilized after field-cooling the lamella from 368 to 100 K in the presence of a magnetic field of 320 Oe that was oriented perpendicular to the lamella, i.e. parallel to the easy axis of Cr_1+δ_Te_2_. The magnetic field was then set to zero and LTEM images were recorded in an under-focus condition (Δ*f* = −1.5 mm). No magnetic contrast is observed when the electron beam is oriented parallel to the lamella [0001] direction, as shown in Fig. 3b. However, a magnetic contrast becomes visible once the lamella is tilted a few degrees away from the impinging electron beam (see schematic in Fig. 3a). Data for a tilt angle of 15° (around the *y*-axis, here denoted as +*α* tilt) are shown in Fig. 3c. The LTEM image shows small circular features that are characterized by dark and bright contrast at their opposite edges. This contrast is reversed for opposite tilt angles (−*α* tilt, see Fig. 3d). Similarly, for a tilt about the *x*-axis (±*β* tilt), the magnetic contrast is rotated by 90° relative to the ±*α*-tilt (see Fig. 3e, f). These observations are characteristic features of Néel-type skyrmions. In the absence of sample tilting the in-plane components of the magnetic texture are aligned either radially outward or inward. Therefore, the deflected incoming electrons do not generate any intensity modulation in the defocused image plane resulting in the absence of any magnetic contrast^37^. Tilt angle-dependent LTEM images of these Néel skyrmions are shown in the SI (see Fig. S14). Figure 3g shows a LTEM image collected at a lower defocus condition (Δ*f* = −0.5 mm), where we see a similar magnetic contrast as for Δ*f* = −1.5 mm, showing that the contrast is characteristic of the spin texture in this range of defocus values of 0.5–1.5 mm. Figure 3h shows the calculated LTEM contrast for two opposite tilt angles of a prototypical Néel-type skyrmion-bubble, as discussed in methods^26^, demonstrating that the objects we observe are clearly Néel-type.

From the XRD analysis it is concluded that the symmetry of the crystal structure corresponds to the *C*_3*v*_ point group which fulfills the symmetry requirements for hosting Néel-type magnetic textures^38^. The LTEM images indicate that the magnetic nanostructure consists of a larger uniformly magnetized core along with a narrow Néel-wall at its periphery. Furthermore, the magnetic nanostructures in our Cr_1+δ_Te_2_ sample do not form a well-ordered hexagonal 2D lattice. For these reasons we propose that the magnetic textures are not only stabilized by the DMI but are also affected by long-range dipole-dipole interactions (DDI)^39^ and can therefore be considered as Néel skyrmion bubbles. The DDI is also known to stabilize skyrmionic bubbles (type-I; typically, Bloch-like)^40^ and topologically trivial bubbles (type-II)^41^. Both of these textures are characterized by an almost uniformly magnetized center-part and a narrow circular domain wall, which surrounds the inner part. Type I and type II bubbles have the same out-of-plane profile and only differ in the in-plane orientation of the magnetic moments. Upon increasing the magnetic field the uniformly magnetized center part that is magnetized opposite to the applied field shrinks, while the domain wall width remains roughly the same.

**Fig. 4:**
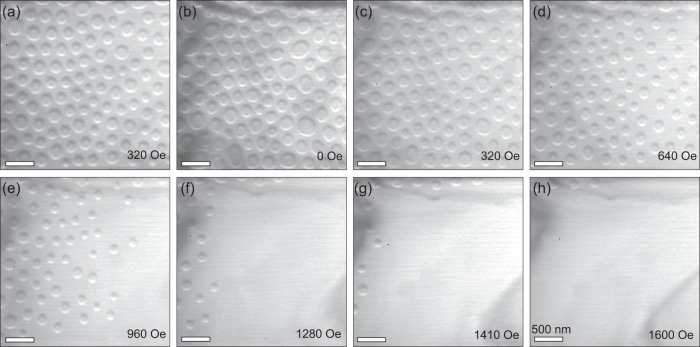
Evolution of Néel-type textures in a thin lamella of Cr_1+δ_Te_2_ as a function of magnetic field at 100 K. **a**–**h** Lorentz transmission electron microscopy images of Néel-type textures collected at different magnetic fields by tilting the lamella 7° away from the [01$\bar{1}$1] direction. These images are acquired at a defocus value of −1.2 mm. The lamella has a thickness of ≈ 75 nm.

In the following, the stability and size-dependence of the Néel skyrmions under variation of the magnetic field and thickness of the lamella is described. At first, we show the results of the magnetic field-induced evolution of the spin textures after field cooling at a field of 320 Oe applied along the [01$\bar{1}$1] direction. Similar results are found for fields in the range of 60-320 Oe. At lower fields the skyrmions are intermixed with cycloidal structures and at higher fields the skyrmion density is lower. Since the magnetic contrast is bigger, the larger is the tilt angle, we show the LTEM images of skyrmions with the electron beam directed along the [01$\bar{1}$1] direction under different magnetic fields at 100 K (Fig. 4), corresponding to a tilt angle of ≈33° with respect to the sample normal [0001]. The initial state in the presence of the field shows the presence of Néel skyrmions as shown in Fig. 4a. Upon reducing the magnetic field to zero the skyrmions remain stable (Fig. 4b), while their average size becomes larger by ≈ 8 %. Further increase of the magnetic field up to 640 Oe (Fig. 4d) leads to a decrease of the density of skyrmions until they start to disappear when the field strength is increased from 960 to 1280 Oe (Fig. 4e–g). Finally, at 1600 Oe almost the whole lamella is in the field-polarized state together with just a few isolated skyrmions (Fig. 4h).

**Fig. 5:**
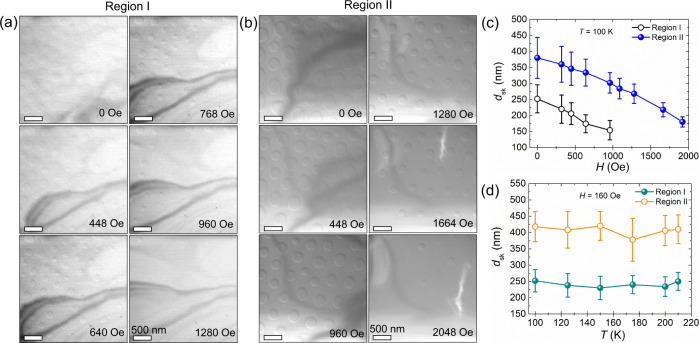
Thickness-dependent evolution of Néel-type textures in a thin lamella of Cr_1+δ_Te_2_ as a function of magnetic field at 100 K. **a**, **b** Lorentz transmission electron microscopy image of Néel-type textures recorded at different magnetic fields for *α* = +6° for lamella thicknesses of **a** ≈ 75 nm (Region I) and **b** ≈ 162 nm (Region II). These images are acquired at a defocus value of −1.5 mm. **c** Skyrmion diameter (*d*_sk_) versus magnetic field (*H*) for Regions I and II. **d** Skyrmion diameter (*d*_sk_) versus temperature (*T*) for Regions I and II. The dark lines correspond to bending contours. The error bars in **c**, **d** represent the standard deviation in the skyrmion diameter.

The field-driven evolution of the skyrmions is studied for two different thickness regions [≈75 nm and ≈162 nm labeled as region I (bright) and region II (dark), respectively]. Following the field-cooling from 368 K in a vertical magnetic field of 64 Oe oriented parallel to the [0001] direction, Néel skyrmions were stabilized in both thickness regions (Fig. 5a, b). The skyrmions are larger in region II than in region I. With increasing magnetic field, skyrmions first disappear at 1280 Oe from region I, while they are still stable within region II, i.e. the skyrmions are considerably more stable within the thicker region until, at a field strength beyond 2048 Oe, all skyrmions have completely been transformed into the field-polarized state. Although in both regions the magnetic objects appear simultaneously under the same magnetic field, their disappearance at a different magnetic field points to a significant thickness dependence of their stability window. In general, the diameter of skyrmions decreases with increasing field. The relation between skyrmion diameter and applied field within both regions is shown in Fig. 5c. An interesting point is that the width of the size distribution at a given field decreases as the field is increased. This is consistent with the role of DDI mentioned earlier. As the central core region shrinks with increasing field, the size of the skyrmions is determined largely by the Néel domain wall width and the influence of the DDI becomes less important. It is clear that the broad size distribution in small fields is governed by DDI rather than by any intrinsic variation of the magnetic properties. LTEM images recorded at temperatures varying from 100–220 K are shown in SI (see Fig. S15). We note that there is no significant temperature-dependence of the skyrmion diameter for this temperature range, as shown in Fig. 5d. This would imply that the magnetization is also insensitive to temperature in this range otherwise, as discussed in Ref. ^26^, the DDI would evolve with temperature. Interestingly, as shown in Fig. 2a, the magnetization for low fields, where we observe the skyrmions, is nearly independent of temperature up to close to the Curie temperature.

In summary, we have found a new crystal structure for the self-intercalated vdW compound Cr_1+δ_Te_2_ (δ ≈ 0.3) which is characterized by the presence of two symmetry inequivalent vdW gaps per unit cell. This structure lacks inversion symmetry which is thereby compatible with the presence of bulk DMI which makes possible the presence of the Néel-type skyrmions that we observe below the Curie temperature of ≈ 200 K. The properties of these Néel spin textures are similar to those reported in PtMnGa^38^ and Fe_3_GeTe_2_^42^ which have crystal structures with the same SGR. We also note that the significant role of DDI is similar to that found in the inverse tetragonal Heusler compounds although these compounds, which have a different SGR, rather host anti-skyrmions and elliptical Bloch skyrmions^26^. Our results provide an example that in vdW materials self-intercalation can generate very complex atomic geometries in otherwise simple crystal structures which opens up new perspectives to create vdW crystals by tuning self-intercalation with novel functionalities.

## Methods

### Structural and magnetic characterization

The X-ray diffraction experiments were carried out using a Gallium Jet X-ray source operated at an acceleration voltage of 70 kV and a power of 200 W emitting Ga-*K*_α_ radiation (*λ* = 1.341 Å) monochromatized by a Montel optics. The intensities of the diffracted peaks were collected with a six-circle diffractometer and a two-dimensional Pilatus 100k pixel detector. Temperature dependent experiments were carried out in a helium cooled Cryovac Konti Micro cryostat. Integrated intensities of the diffraction peaks used for the structure analysis were collected by transverse (theta-) scans which were subsequently subject to instrumental correction factors (Lorentz-, polarization, effective area, absorption) to derive the structure factor magnitudes. The temperature and field (*H*) dependent magnetization (*M*) measurements of the single crystal of Cr_1+δ_Te_2_ (δ ≈ 0.3) were carried out with a SQUID-VSM [MPMS3, Quantum design].

### Transmission electron microscopy

For the transmission electron microscopy (TEM) investigations, several lamellae from the same single crystal of Cr_1+δ_Te_2_ were prepared by Focused Ion Beam (FIB) Ga^+^ ion milling [TESCAN GAIA3 operating at 30 kV ion-beam energy] using standard lift-out procedures. Final polishing of the lamellae was performed with lower Ga^+^ ion-beam energies (5 kV) to reduce the thickness of any amorphous surface layers. The thickness, *t*, of the lamella was measured using Energy Filtered TEM (EFTEM) by the log-ratio method from the relationship, *t* = *λ* ln (*I*_t_/*I*_0_), where *λ* is the total inelastic mean free path of the electrons, and* I*_t_ and *I*_0_ are the total and zero-loss intensities in the EELS spectrum, respectively^43^. The error in measuring *t* is ~10 %. Magnetic textures were investigated by TEM [FEI TITAN 80-300] in the Lorentz mode operated at an accelerating voltage of 300 kV using a GATAN double-tilt sample holder which is capable of varying the temperature between 100 and 368 K. A vertical magnetic field was applied to the lamella within the TEM column by passing currents through the coils of the objective lens and a Lorentz mini-lens was used for imaging.

### Calculation of Lorentz TEM image contrast

We have calculated the Lorentz TEM images based on the method in Ref. ^26^. The density of transmitted electrons determines the contrast at position $(x,y)$ as

$$I\left(x,y\right)=\int {{\exp }}\frac{-[{(x-d\cdot {m}_{y}(x^{\prime} ,y^{\prime} )-x^{\prime} )}^{2}{+\left(y+d\cdot {m}_{x}\left({x}^{{\prime} },{y}^{{\prime} }\right)-{y}^{{\prime} }\right)}^{2}]}{{a}^{2}}{{{{{\rm{d}}}}}}x^{\prime} {{{{{\rm{d}}}}}}y^{\prime}$$

The electron beams are approximated as Gaussian functions with a smearing factor *a* = 1 nm. Due to the Lorentz force, the averaged magnetization $m(r)$ along the layers leads to a transverse deflection of the incoming electron beams. The maximum deflection is controlled by *d* = 0.5 nm.

## Supplementary information

Supplementary Information

## Data Availability

The data that support the findings of this study are available from the corresponding author upon request.

## Code availability

The source code for the calculations performed in this work is available from the corresponding authors upon request.
