# Supplementary material for: Observation of Néel-type skyrmions in acentric self-intercalated Cr1+δTe2
Source: Nat Commun. 2022 Jul 8;13:3965. doi: 10.1038/s41467-022-31319-y (PMC9270380; doi:10.1038/s41467-022-31319-y)
Supplement: Supplementary file 1 — Supplementary Information [file 41467_2022_31319_MOESM1_ESM.pdf]

Supplementary Information

Observation of Néel-type skyrmions in acentric self-intercalated $Cr_{1+\delta}Te_2$

Rana Saha[1], Holger L. Meyerheim[1], Börge Göbel[2], Binoy Krishna Hazra[1], Hakan Deniz[1], Katayoon Mohseni[1], Victor Antonov[3], Arthur Ernst[1,4], Dmitry Knyazev[1], Amilcar Bedoya-Pinto[1,5], Ingrid Mertig[2] & Stuart S. P. Parkin[1] ✉

[1]Max Planck Institute of Microstructure Physics, Weinberg 2, Halle (Saale) 06120, Germany

[2]Institute of Physics, Martin Luther University, Halle-Wittenberg, Halle (Saale) 06120, Germany

[3]G. V. Kurdyumov Institute for Metal Physics, National Academy of Science of Ukraine, 03142 Kiev, Ukraine

[4]Institute for Theoretical Physics, Johannes Kepler University Linz, Altenberger Strasse 69, A-4040 Linz, Austria

[5] Instituto de Ciencia Molecular, Universidad de Valencia, Catedrático José Beltrán 2, Paterna, 46980 Spain

✉stuart.parkin@mpi-halle.mpg.de

A. X-Ray Diffraction Analysis

A1. Data collection and evaluation:

X-ray diffraction experiments were carried out using a Ga-metal jet x-ray source (λ=1.34 Å) and a six-circle diffractometer equipped with a two-dimensional pixel detector and a helium cooled cryostat. Integrated reflection intensities were collected under grazing incidence (μ=1°) of the incoming beam by transverse (theta-) scans keeping the detector position fixed. We find that the superstructure is related to bulk-like unit cell by a (2×2×2) super-periodicity since both in-plane reflections, H and K, are (simultaneously) at half order positions in addition to the half order out of plane reflections L. The observation of a simultaneous appearance of half-order reflections such as e.g. (1/2 ½ L) could also originate from a (2×1) in-plane superstructure which

is twinned by the presence of additional symmetries such as an (apparent) additional vertical mirror plane. While this is frequently observed in the case of thin films adsorbed on high-symmetry substrates (e.g. 6mm plane point group symmetry) like graphene or MoS_2, in the present case this can be excluded because the symmetry of the diffraction pattern would be six-fold rather than threefold as observed.

For the (2×2×2) superstructure in total 158 symmetry independent reflections were collected. The data set consists of 36 integer order reflections related to the average (1×1×1) structure and 122 fractional (half-order) reflections related to the full (2×2×2) superstructure. The latter are several orders of magnitude weaker than the integer order reflections as they originate from the *difference* between the Cr-intercalation structures within the two inequivalent van der Waals (vdW) gaps as shown in Fig.1 of the main text. By contrast, the integer order reflections originate from the average structure with contribution from all atoms, primarily from Te atoms with large scattering amplitude.

Uncertainties (1σ) of the measured reflection intensities were derived by the quadrature sum of the statistical and the systematic uncertainty, the latter derived from the reproducibility of symmetry equivalent reflections. Since only reflections (HKL) with indices L>0 were collected the plane group symmetry P3m1 was used for symmetry averaging. We derived an average agreement of 14% for the main reflections which represents a reasonable value and confirms the trigonal symmetry of the diffraction pattern, i.e. sample twinning leading to a higher six-fold symmetry can be ruled out.

A2. Structure refinement and model:

The structure analysis was carried out by least squares refinement of the calculated structure factor magnitudes ($|F_{calc}(HKL)|$) to the observed ones ($|F_{obs}(HKL)|$). The latter were derived from the integrated intensities by applying instrumental correction factors (Lorentz-,

polarization factor as well as effective area). As the number of weak superstructure reflections greatly exceeds the number of the strong integer order reflections, we used an un-weighted refinement procedure, i.e. all $|F_{obs}(HKL)|$ were treated with the same weight, i.e. the different uncertainties associated with each reflection was neglected. Here, the un-weighted residuum (Ru) is minimized which is defined as: $Ru = \sum_{HKL} ||Fobs(HKL)| - |Fcalc(HKL)||/\sum_{HKL} |Fobs(HKL)|$

For the refined structure model, we obtained for Ru=0.11, which given the large number of weak reflections represents an excellent value.

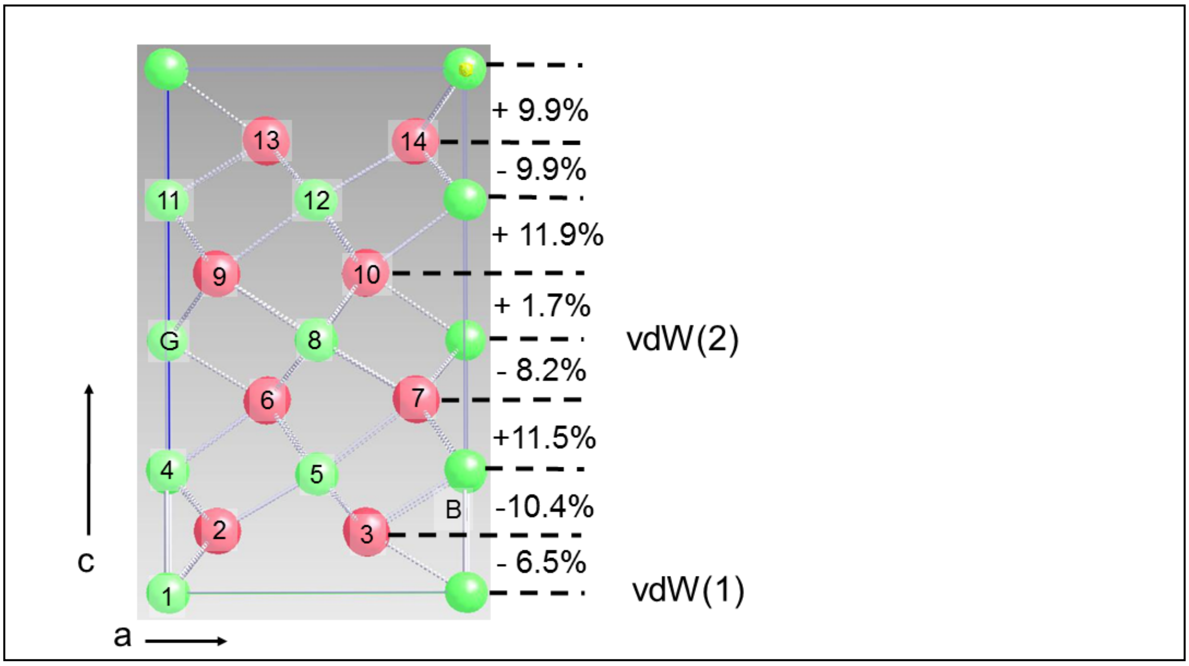

Fig. S1: Model of the $Cr_{1+\delta}Te_2$ (2×2×2) structure viewed parallel to the [01$\bar{1}$0] direction to emphasize the atomic vertical relaxations. Atoms are labelled as in Figure 1 in the main text. Relaxations of the interlayer distances normalized to the bulk value (0.125 lattice units=1.496 Å) are given on the right in percent. Uncertainty is about 5 percentage points. Vertical atomic shifts involve a symmetry reduction from SGR P$\bar{3}$m to P3m1.

The most important result is that the vdW gaps [vdW(1) and vdW(2) in Figure 1(e) of the main text] are inequivalent. This is due to the occupation of different sites by Cr atoms within the two inequivalent vdW gaps. Figure S1 shows the structure model derived from the least squares fit. For simplicity the view parallel to the [10$\bar{1}$0] direction is shown to focus on the atomic relaxations. The two symmetrically independent vdW gaps are labelled by vdW(1) and vdW2.

In vdW(1) only the Cr atom labelled by (1) is located, while in vdW(2) three symmetry independent atoms are located labelled by (8), (G) and (H), the latter located behind atom (8) at (x, y, z)≈(½ ½ ½) (see also Figure 1e of the main text). The sites in vdW(2) are occupied by a probability considerably less than 100% (60±20%) while the Cr site (1) in vdW(1) is fully occupied. This disorder can be interpreted by the presence of domains, in which at y=1/2 either site (G) or (H) is occupied while a vacancy exists at the other, respectively. Similarly, for site (8) every other unit cell is occupied on average which allows to construct a model where for instance in one domain all sites (8) are occupied in neighboring unit cells along the a-axis, but an array of vacancies exist in another domain. This disorder model involving domains of particular occupied sites and vacancies requires that the domain size is smaller than the coherence length of the x-ray beam (ξ≈50-100 nm), as otherwise extra reflections would appear, which -however- are not observed. In conclusion it should be kept in mind that the model outlined here for the (2×2×2) unit cell corresponds to the structure averaged over the domain size. Only for this average structure the symmetry is found to be P3m1, on a scale lower than the domain size it is very likely to be even lower.

The in-equivalence of the vdW gaps involves vertical relaxations of the atoms within the structure. Relaxations of the interlayer distances relative to the unrelaxed bulk value (0.125 lattice units=1.496 Å) are listed on the right. Uncertainties are in the 5% point regime. Relaxations along the c-axis reduce the symmetry from the centrosymmetric SGR $P\bar{3}2/m$ of the average (1×1×1) structure to P3m1. It is also conceivable that the symmetry of the average structure is even lower (P1) as Ru adopts the absolute minimum of Ru=0.116 if all atoms are allowed to relax independently, but the differences between the z-parameters of atoms within one layer differ by 0.007 lattice units (LU) at most which is considered as not significant. We emphasize that the atomic relaxations are decisive for obtaining a good fit. Keeping the

interlayer distances at the bulk value of 0.125 LU, the fit quality significantly increases by more than 30% relative to the best fit.

A3. Temperature dependent XRD:

In a further experiment we have investigated the temperature dependence of the $Cr_{1+\delta}Te_2$ structure. We have collected three data sets at 295 K, 50 K and 4 K using a helium cooled Cryovac Konti Micro cryostat. For each data set about 35 symmetry independent reflections were collected reducing to approximately 23 integer order reflections and 6 fractional order reflections by symmetry averaging. The quantitative analysis using least squares refinement indicates that there are no temperature-dependent modifications of the structure, apart from a reduction of the atomic displacement parameters (ADP's), commonly referred to as the Debye-Waller factor, which has a thermal and a static contribution[1].

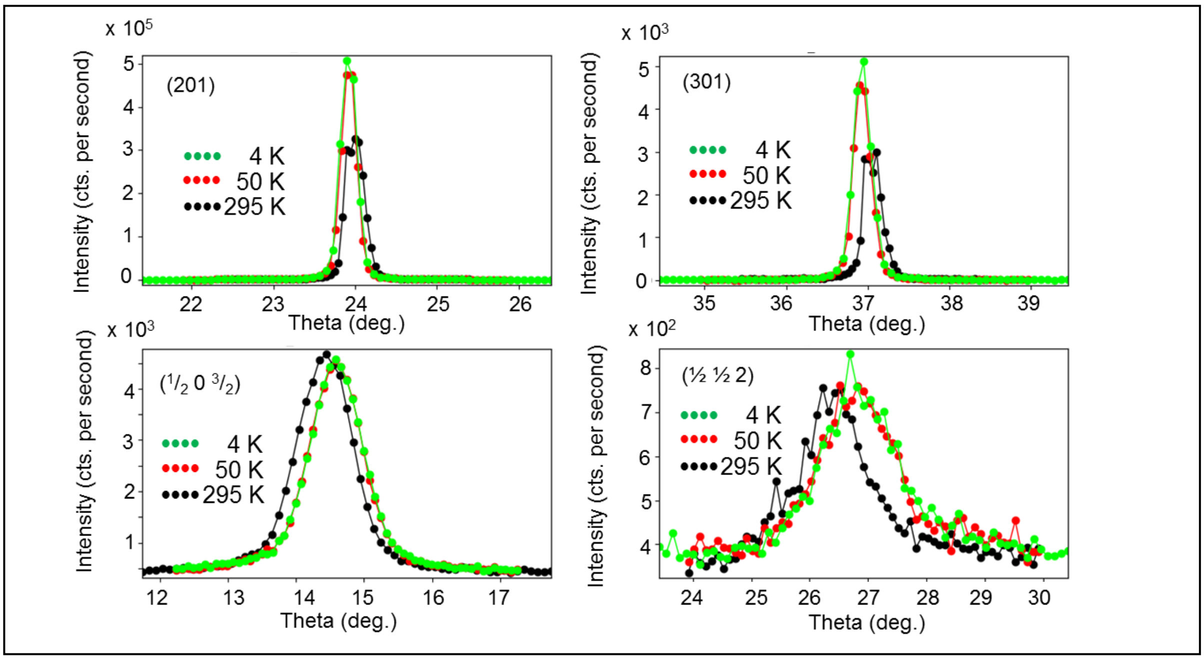

Fig. S2: Temperature dependence of main (integer) and fractional order reflections. Theta scans of integer (upper panels) and fractional order (lower panels) reflections collected at 295 (black), 50 (red) and 4 K (green). Upon sample cooling from 295 K to 4 K the integer order reflection intensities increase by about 50%, while for the fractional order reflections the intensity remains nearly constant. This is because the latter originate only from the difference

between the intercalation structures within the two inequivalent vdW gaps. Upon cooling the intensity increase related to the freezing of the thermal disorder tends to cancel out.

In Figure S2 we show transverse (theta-) scans across several integer (upper panels) and half order (lower panes) reflections versus temperature (295, 50 and 4K). While there is a strong intensity increase of almost 50% in the case of the integer order reflections, the intensity of the half order reflections remains almost unchanged. At first view this appears as surprising, as one would expect that upon cooling the reduced thermal disorder would lead to an increase of the reflection intensity. The strongly suppressed sensitivity of the half order reflections to thermal disorder can be explained by recalling that the half order reflections originate from the difference between intercalation structures within the two vdW gaps. In other words, at the half order reflections there is an anti-phase scattering relationship between the amplitudes scattered by the Cr atoms located in vdW(1) and vdW(2). In the limit of identical structures in vdW(1) and vdW(2) these reflections would completely disappear. The anti-phase scattering and the occupation of both vdW gaps by Cr – albeit at different sites and different concentration- is responsible for the extraordinary weakness of the superstructure reflections as compared to those with integer reflection indices (see Fig. 1a of the main text). There is only a small contrast between the charge density in vdW(1) and vdW(2). Typical two-fold superstructures commonly found for intercalation structures so far are characterized by a fully occupied site followed by a vacancy, for which the contrast is at a maximum and the reflection intensities are correspondingly high and easier to observe. In the present case another consequence of the anti-phase scattering is also that upon sample cooling, thermal vibrations of the Cr atoms in both vdW gaps are reduced by approximately the same amount so that the gain in scattering amplitude by the reduced "Debye-Waller" factor almost cancels out.

B. Energy Dispersive X-ray (EDX) Spectroscopy analysis

The chemical composition of the crystal was investigated by energy dispersive x-ray spectroscopy (EDX) analysis at different places on the crystal and found similar compositions indicating a homogeneous sample. The ratio between the Cr and the Te concentration was determined to be $Cr_{1.16}Te_2$ which is less than that derived by XRD, where we find approximately $Cr_{1.3}Te_2$. We note however, that the discrepancy might originate from the fact that EDX probes the total concentration averaged over the whole sample volume, while XRD probes the coherent fraction only, i.e. the fraction of the sample volume which crystallizes in the (2×2×2) lattice and thus contributing to the diffraction.

Table 1: Parameters of the EDX analysis of the $Cr_{1+\delta}Te_2$ sample.

Element	Atomic No.	Mass (%)	Mass Norm. (%)	Atom (%)	Absolute Error (%)	Relative Error (%)
**Carbon**	6	1.03	1.06	8.21	0.23	22.70
**Chromium**	24	18.34	18.88	33.65	0.53	2.87
**Tellurium**	52	77.78	80.06	58.14	2.25	2.89
	**Sum**	97.16	100.00	100.00		

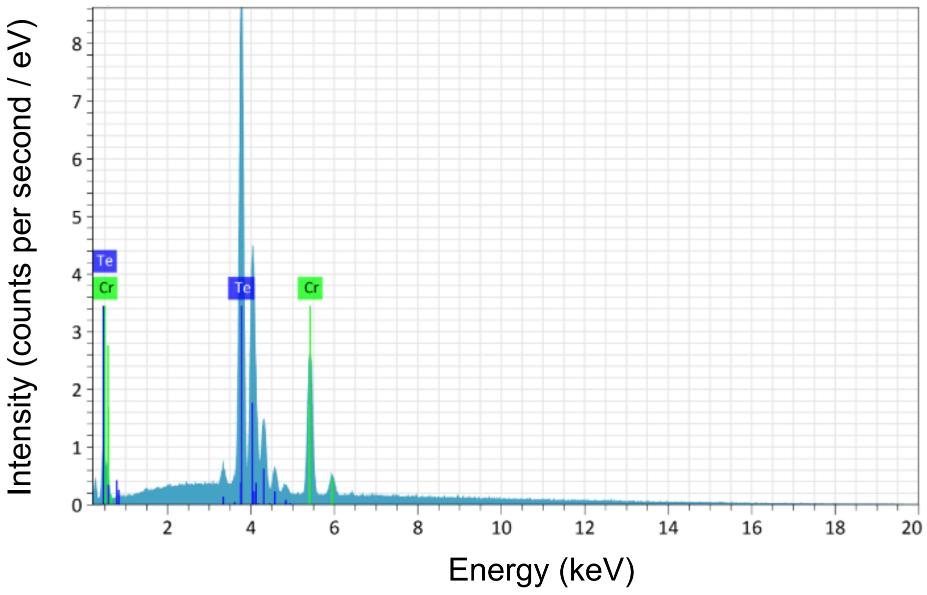

Fig. S3: Energy Dispersive X-ray (EDX) Spectroscopy. EDX spectra taken from the surface of a single crystal of $Cr_{1+\delta}Te_2$ using scanning electron microscopy at an energy of 20 keV.

C. X-ray absorption (XAS) and X-ray circular magnetic dichroism (XMCD)

The XAS/XMCD experiments were carried out a $Cr_{1+\delta}Te_2$ crystal cleaved in the preparation chamber of the high-field vector magnet end station HECTOR at BOREAS beamline (ALBA, Barcelona), under ultra-high vacuum conditions (p=10^{-10}mbar). The x-rays were generated by an APPLE-II type undulator with full circular or linear polarization. The absorption signal was measured in the total electron yield (TEY) mode monitored by the sample-to-ground drain current. The sample absorption signal was normalized by the primary photon flux determined by the TEY signal on a freshly evaporated gold mesh which was placed between the las optical element and the sample. The drain current signals were detected by a Keithley 428 current amplifier. The sample temperature was varied between 2 K and 300 K as measured on the cold finger of the cryostat. A magnetic field of up to 6 T was applied along the beam direction in order to magnetically polarize the sample during the XMCD experiments, and the XMCD signal was evaluated as the difference of the XAS signal in the two opposite helicities.

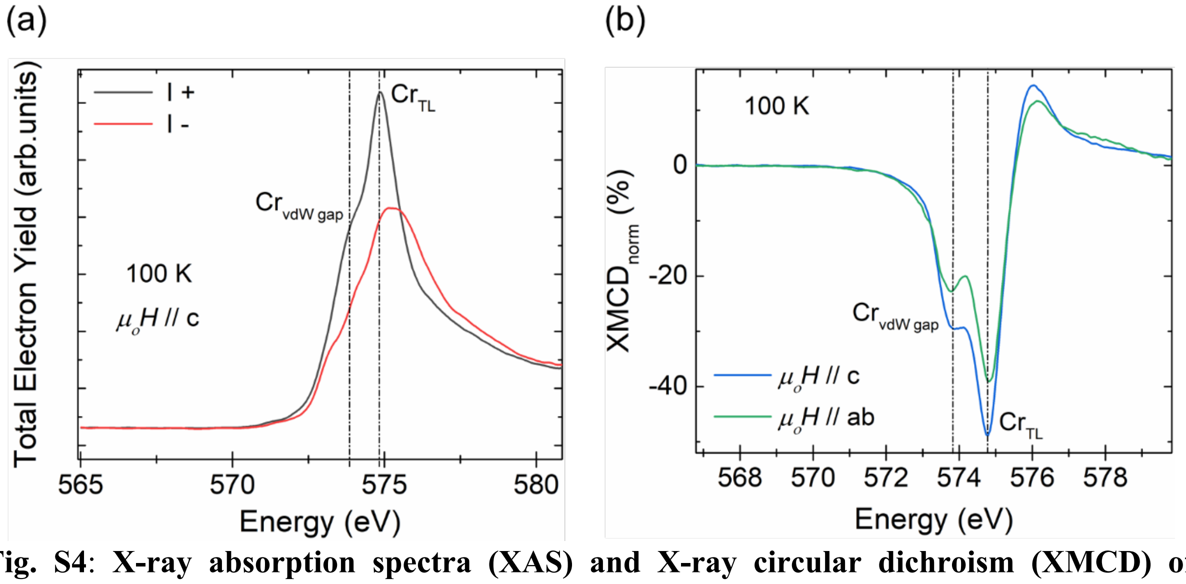

Fig. S4: **X-ray absorption spectra (XAS) and X-ray circular dichroism (XMCD) of $Cr_{1+\delta}Te_2$ crystal. (a)** XAS collected by total electron yield (TEY) and (b) XMCD of the $Cr_{1+\delta}Te_2$ crystal collected at 100 K in the presence of 6 T magnetic field showing two absorption peaks corresponding to different Cr sites in the crystal, namely at 574.8 eV (Cr in the Te-Cr-Te triple layers, TLs) and 573.8 eV (Cr in the vdW gap), respectively (see also Fig. S6 for theoretical analysis). The XMCD spectra in (b) at different incidence angles show that both Cr

(TL) and Cr (vdW gap) peaks contribute to the anisotropy of the magnetic moment, along the easy axis (//c) and hard axis (//ab).

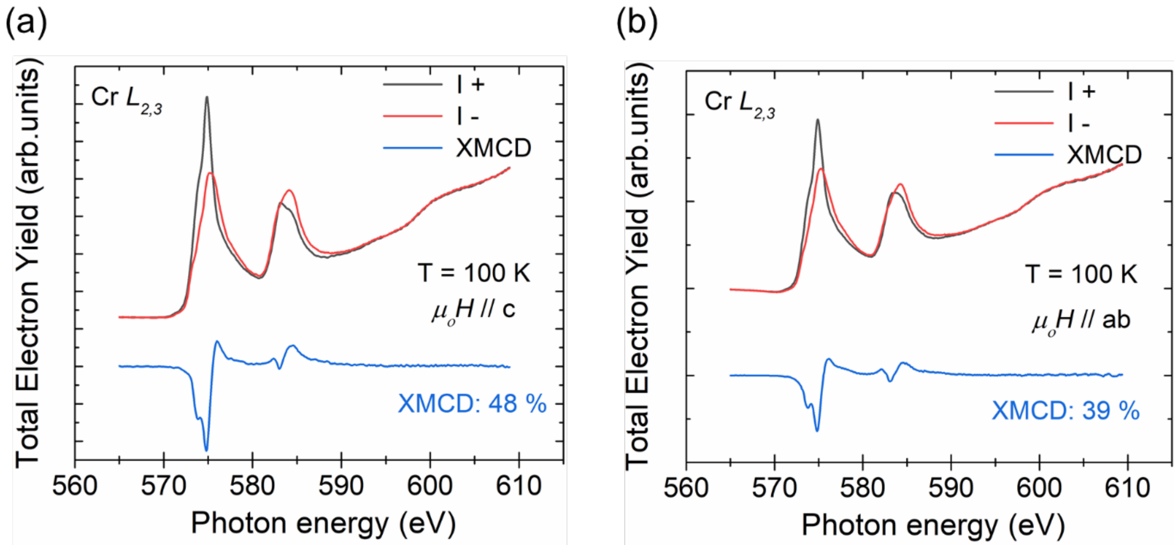

Fig. S5: X-ray absorption spectra (XAS) with different photon helicities and the corresponding X-ray magnetic circular dichroism (XMCD) monitored in the vicinity of the Cr $L_{2,3}$ edge under an applied magnetic field ($\mu_0 H$) of 6 T. XAS and XMCD with $\mu_0 H$ oriented along the out-of-plane (normal incidence, NI) **(a)** direction and along **(b)** the in-plane direction (grazing incidence, GI). XMCD signal is higher for the NI case, corresponding to an out-of-plane easy axis, in agreement with volumetric magnetization measurements.The background of the XAS spectra arises from the Te-$M_{4,5}$ edge.

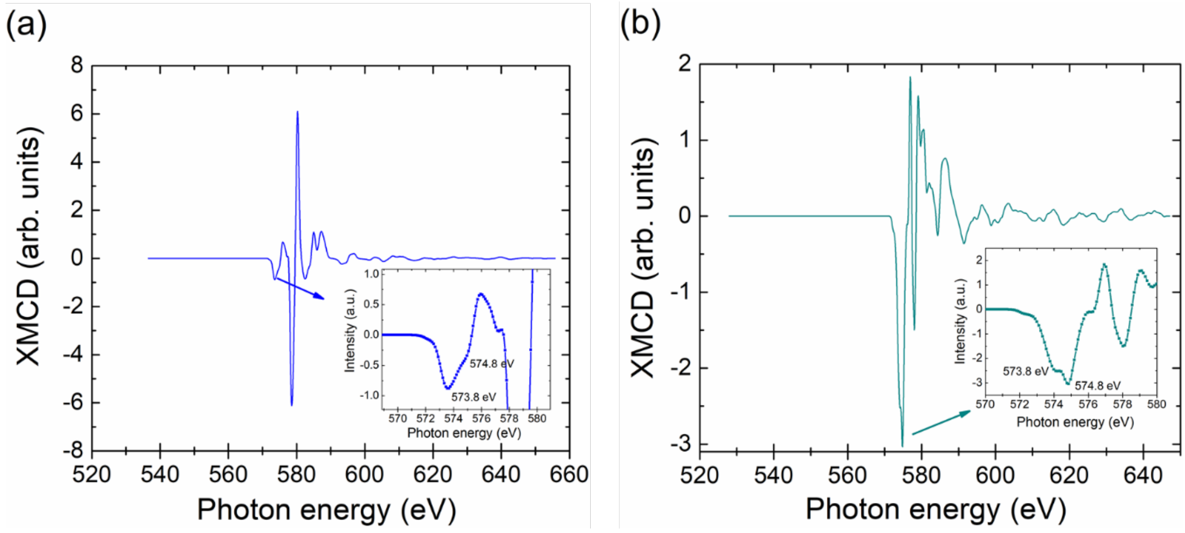

Fig. S6: **Simulated XMCD spectra within the linear muffin-tin orbital (LMTO) method.** (a) Without intercalated Cr in $CrTe_2$ and (b) with intercalated Cr atoms in $Cr_{1.3}Te_2$. In the former case the shoulder at 573.8 eV is missing.

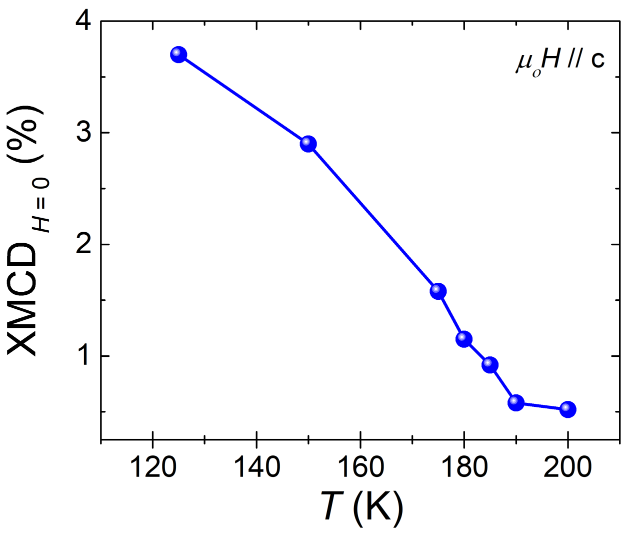

Fig. S7: Remanent magnetic moment from XMCD. Temperature dependence of the remanent magnetic moment (XMCD, H=0), at normal incidence (H//c).

D. First-principles calculations

In order to analyze the absorption and XMCD spectra quantitatively, we have carried out first-principles calculations of the electronic and magnetic structure of $Cr_{1+\delta}Te_2$ utilizing the structural model derived from the XRD experiment. Calculations were carried out using a self-consistent Green function method within the multiple-scattering theory, which is specially designed for alloys and systems with other imperfections[2]. Disorder effects were taken into account within the coherent-potential approximation (CPA)[3, 4]. In combination with a self-interaction correction (SIC) approach[5], this method enables an efficient determination of the valence state (or oxidation state) of ions[6, 7]. The nominal valence, N_{val} , is defined as: $N_{val} = Z - N_{core} - N_{SIC}$,where Z is the atomic number, N_{core} is the number of core (and semicore) states and N_{SIC} is the number of self-interaction corrected states. The ground state valence is the one defined by the ground state energy. By probing various SIC state configurations it has to be tested which states need to be corrected in order to find the ground state and the ground state valency. For both Cr atoms in $Cr_{1+\delta}Te_2$ we found the valence to be equal to 3+ with the self-interaction corrected states $d_{x^2-y^2}, d_{z^2}, d_{xy}$.

To investigate the impact of intercalated Cr atoms on the electronic and magnetic structure of $Cr_{1+\delta}Te_2$ we compared two cases: $CrTe_2$ and $Cr_{1+\delta}Te_2$ with $\delta=0.3$. Magnetic order was determined using the magnetic force theorem analysing the calculated spin wave spectra (see Fig. S8). In $CrTe_2$ there is only one magnetic sub-lattice, i.e. Cr atoms are located only within the TLs. Therefore, there is only one spin wave mode as shown in Fig. S8(a). The magnon energy of this mode is positive throughout the whole Brillouin zone (BZ) revealing a stable ferromagnetic ground state. Our calculations also reveal that in this case the magnetization of the Cr moments lies in the plane which is related to the weak interlayer coupling between the Cr atoms. This situation significantly changes if Cr atoms are also located within the vdW gaps. In this case, the spin wave spectrum exhibits two modes, one being characterized by a negative magnon energy. In addition, the minimum of the magnon dispersion relation is located between the Γ- and the A high symmetry points of the BZ. This can be due to a non-collinear magnetic order in the system. Analysing the calculated exchange coupling constants we found that the Cr magnetic moments in the TLs remain ferromagnetically coupled while the Cr spins in the vdW gaps are non-collinear with respect to the spin of Cr in the TL. The presence of Cr atoms in the vdW gap also leads to the out-of-plane magnetization of Cr moments in the TLs.

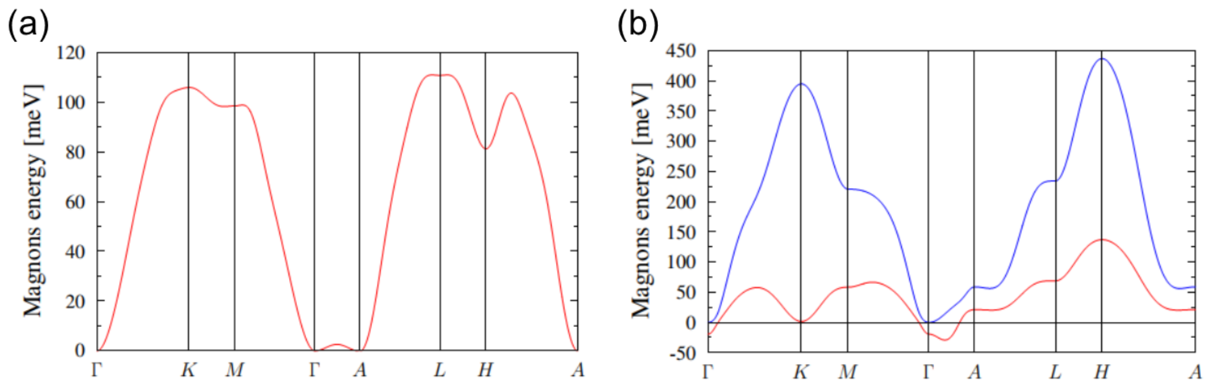

Fig. S8: Calculated magnon dispersion spectra in $CrTe_2$ (a) and $Cr_{1.3}Te_2$ (b). The two lines in (b) represent two magnon modes corresponding to two magnetic sub-lattices in $Cr_{1.3}Te_2$, namely Cr in the TLs and Cr in the vdW gaps. Magnon spectra are calculated using a virtual crystal approximation for the Heisenberg model[8]. The exchange coupling parameters entering the Heisenberg Hamiltonian were computed using a magnetic force theorem as it is

implemented in the coherent potential approximation. In (a) the magnon energies are positive only, therefore, the magnetic ground state is ferromagnetic. Low magnon energies along the Γ-A direction of the BZ in also reveal a weak magnetic coupling between Cr layers in different TLs in $CrTe_2$. Negative energies are observed in (b) and the absolute minimum is located between two high symmetry points within the BZ. These observations indicate on non-collinear magnetic order in the system.

Figure S9 shows the calculated DOS for $CrTe_2$ (a) and $Cr_{1.3}Te_2$ (b), respectively. In the first case the Cr magnetic moment is 2.87 μ_B, while in $Cr_{1.3}Te_2$ the Cr moments in the TL's and in the vdW positions were found to be 2.98 μ_B and 2.45 μ_B, respectively. However, due to the non-collinear magnetic order the net magnetic moment (μ_z) was found to be 2.67 μ_B.

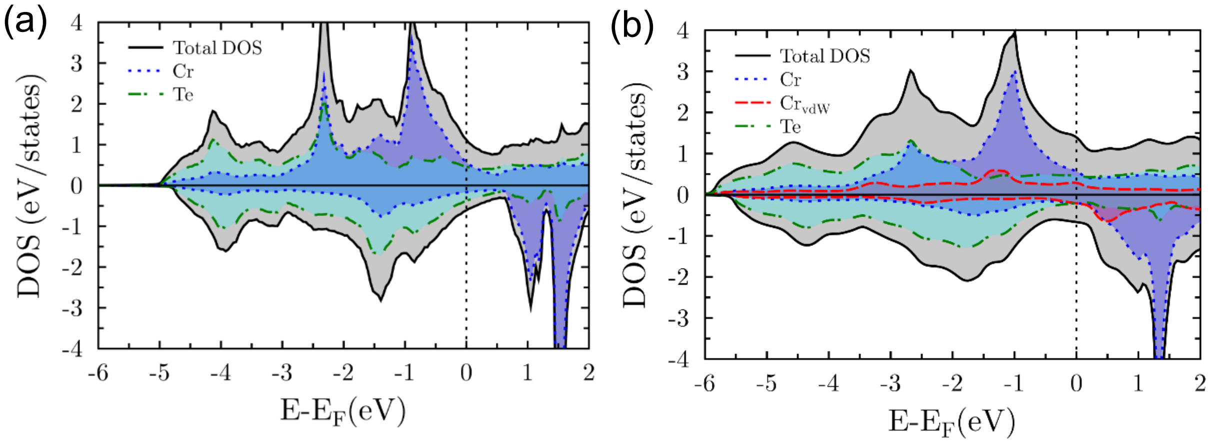

Fig. S9: Calculation of the density of states (DOS) for $Cr_{1+\delta}Te_2$. Calculated atomic and spin resolved DOS of $CrTe_2$ (a) and $Cr_{1.3}Te_2$ (b). Positive (negative) DOS indicate the DOS for spin up (spin down) channels. The presence of Cr in the vdW gaps increases the hybridization of the valence states in $Cr_{1.3}Te_2$ (the valence band width in $Cr_{1.3}Te_2$ is about 1 eV larger than in the case of $CrTe_2$). The strong hybridization between 3*d* states of the Cr atoms located in the TLs and the vdW gaps as well as the hybridization between Cr and Te states is responsible for the non-collinear magnetic structure.

In addition, XAS and XMCD spectra (Fig. S6 and S10) were simulated within a linear muffin-tin orbital (LMTO) method[9-11]. Fig. S10 shows calculated XAS & XMCD spectra for both $CrTe_2$ and $Cr_{1+\delta}Te_2$. XAS spectra are shown atomically resolved separately for the left and right

circular polarized light. The calculations demonstrate clearly that the shoulder in the experimental XMCD spectra at 573.8 eV originates from Cr atoms in the vdW gap.

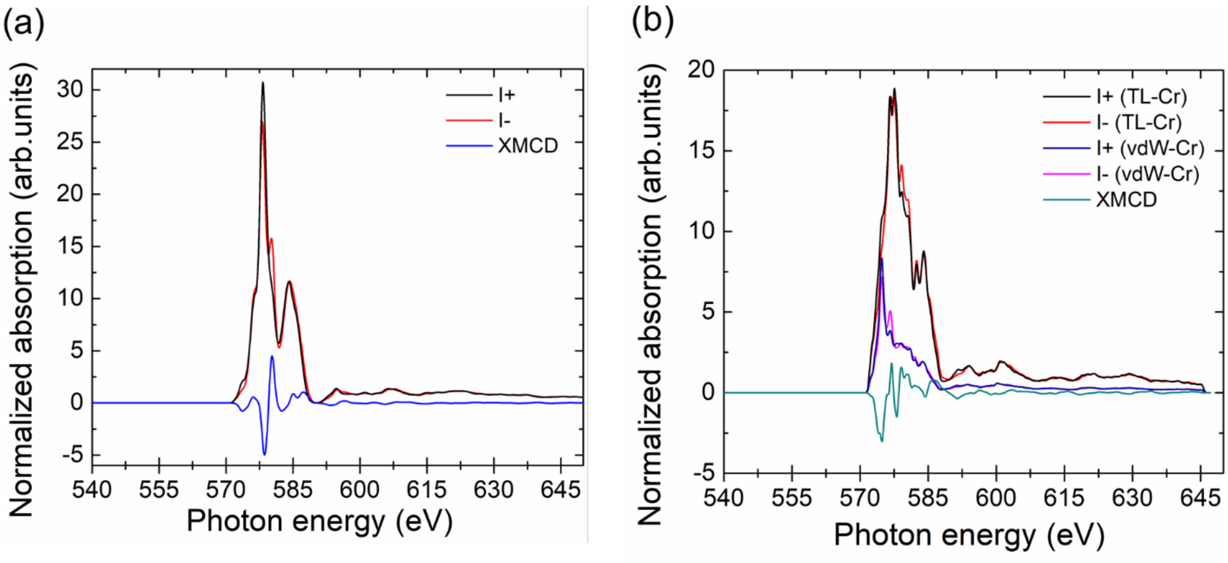

Fig. S10: Calculation of X-ray absorption spectra (XAS) and corresponding X-ray magnetic circular dichroism (XMCD) for $Cr_{1+\delta}Te_2$. Simulated XAS and XMCD spectra within a linear muffin-tin orbital (LMTO) method for (a) $CrTe_2$ and (b) $Cr_{1.3}Te_2$.

E. Temperature-dependent magnetic properties

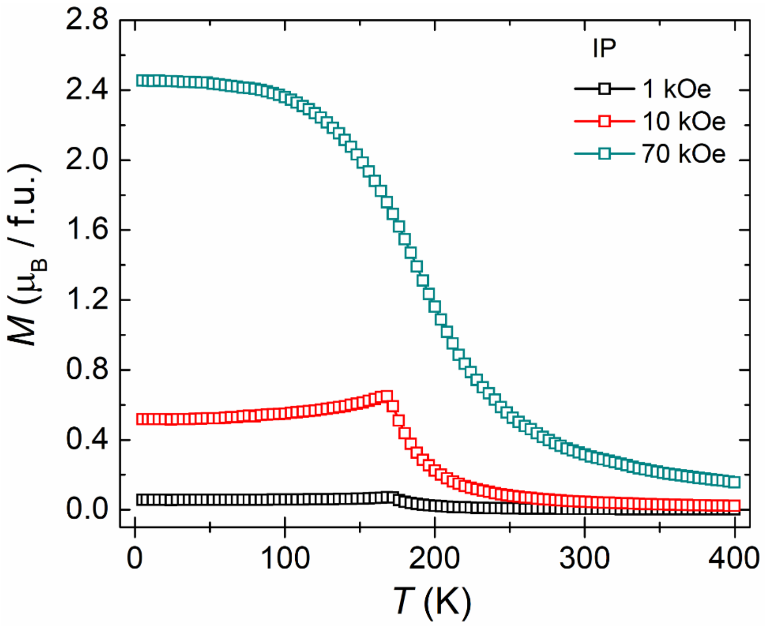

Fig. S11: Magnetization as a function of temperature. Temperature dependence of the magnetization of $Cr_{1+\delta}Te_2$ for different magnitudes of the magnetic field aligned within the sample plane (i.e. perpendicular to [0001]).

F. Magnetotransport properties

The Hall resistance was measured on a Hall bar device prepared by Ga^+ Focused Ion Beam (FIB) from a single crystal of $Cr_{1+\delta}Te_2$. A clear signature of anomalous Hall resistivity was observed below 200 K (Fig. S12a). Clearly, the anomalous Hall resistivity data does not resemble that of the isothermal magnetization data in the same temperature range (Fig. S12b). The additional contribution to the anomalous Hall resistivity, the Topological Hall-effect, indicates the presence of non-collinear spin textures[12, 13]. In Fig. S12c, after subtracting a contribution to the anomalous Hall resistivity in proportion to the measured magnetization, a resulting topological Hall resistivity appears as two peaks of opposite sign with changing polarity of the magnetic field. This study correlates with our findings of Néel-type magnetic textures that are directly imaged by Lorentz transmission electron microscopy (LTEM).

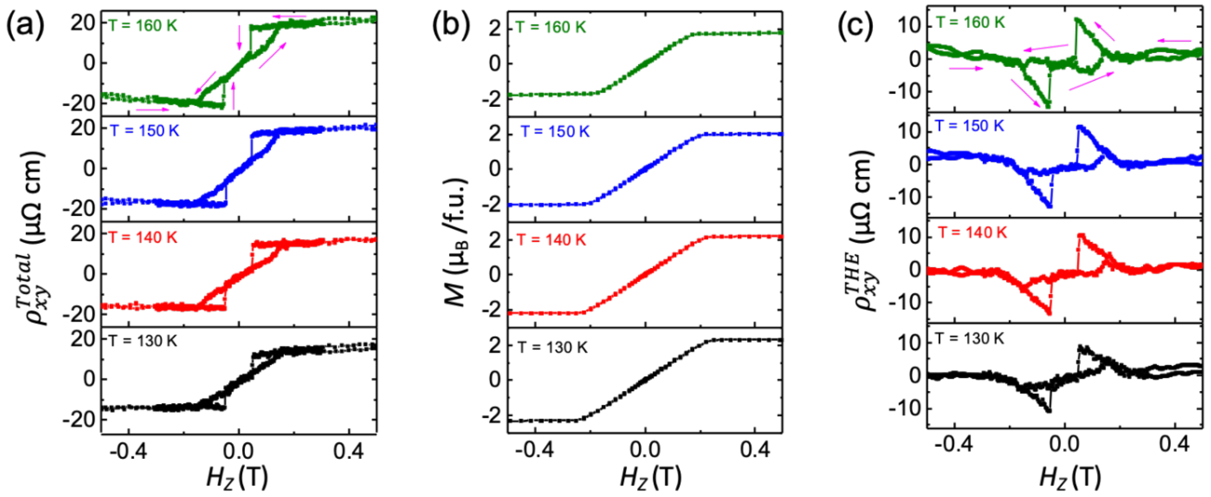

Fig. S12: Magneto-transport properties of $Cr_{1+\delta}Te_2$. (a) Total Hall resistivity data containing anomalous and topological contributions (ρ_{xy}^{Total}) [after subtracting the ordinary Hall resistivity], (b) Field-dependent magnetization data at different temperatures and (c) Topological Hall resistivity (ρ_{xy}^{THE}) at different temperatures.

G. Lorentz transmission electron microscopy (LTEM)

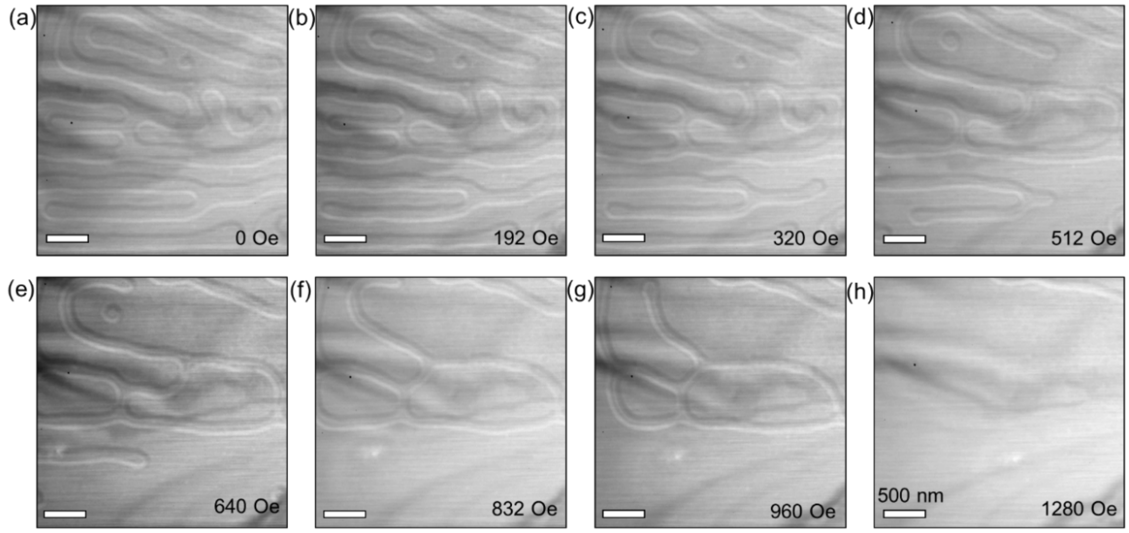

Fig. S13: Evolution of cycloidal states versus magnetic field at 100 K after zero-field-cooling (ZFC) in $Cr_{1+\delta}Te_2$. (a-h) LTEM micrographs of cycloidal states collected at different magnetic fields by tilting the lamella by 11° away from the [0001] direction. Images were collected at a defocus of -1.5 mm. The lamella has a thickness of approximately ≈ 75 nm.

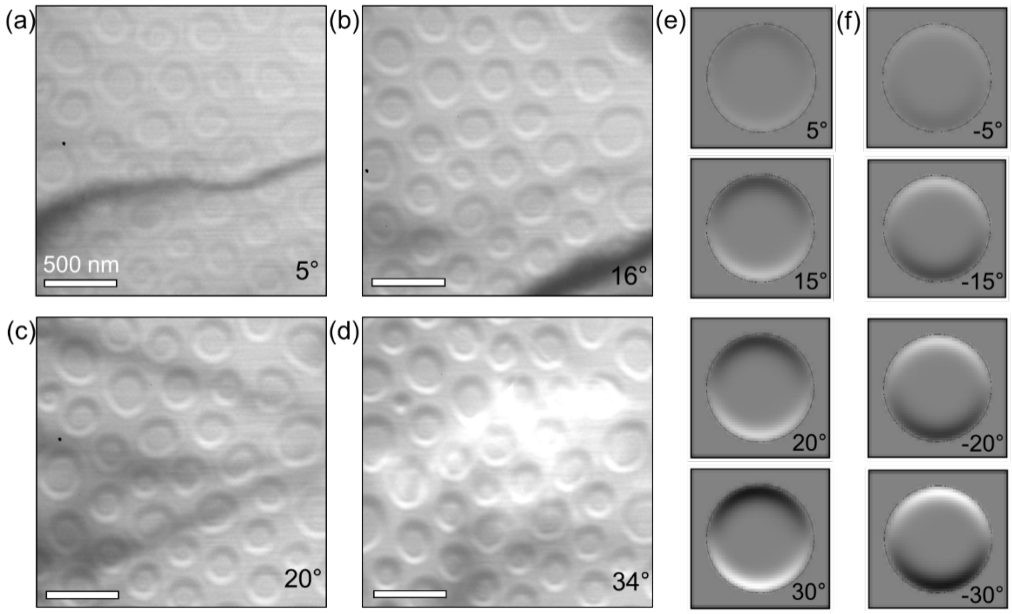

Fig. S14: Tilt-angle (α) dependent evolution of the magnetic contrast of Néel-type skyrmions in $Cr_{1+\delta}Te_2$ at 100 K. (a-d) Lorentz transmission electron microscopy (LTEM) micrograph recorded under different tilt-angles relative to the [0001] direction in zero magnetic field at 100 K. The lamella has a thickness of ≈ 75 nm. Images were recorded at a defocus of -1.5 mm. **(e-f)** Calculated LTEM contrast of Néel skyrmion bubble for a constant defocus under ± α tilt-angles.

Here we discuss the tilt-angle (α) dependent contrast of the Néel-type skyrmions observed in the LTEM images. Without tilting the in-plane components of the magnetic texture which are aligned either radially outward or inward, deflect the incoming electrons so that there is no intensity modulation in the defocused image plane resulting in the absence of any magnetic contrast. By contrast, upon tilting there is a Lorentz force induced asymmetric deflection of the electrons from the projection of magnetization on either side of the tilted textures. As a result, in one side there will be deflected electrons converging thereby generating bright contrast, while on the other side the diverging nature of deflected electrons will produce dark contrast.

After the field cooling process under an applied field of 320 Oe oriented along the [0001] direction, the magnetic field was reduced to zero followed by the data collection of the tilt-angle dependent images at 100 K sample temperature. The experiments were carried out at zero field since this ensures that only the effect of α on the image contrast is explored. Images are shown in Fig. S14a-d for different values of α as labelled in the panels. At α= 5° the contrast is quite weak, but is gradually increases with increasing α from 16° up to 34°. The enhanced magnetic contrast with increasing α is due to the increasing in-plane magnetic component of the spin textures resulting in an enhanced deflection of the transmitted electrons owing to the Lorentz force, the latter being directly proportional to the in-plane magnetic induction created by the specimen[14]. This experiment further provides evidence that the observed magnetic textures are indeed of Néel-type. LTEM image contrast (see Fig. S14 e-f) was calculated for a Néel-type skyrmion bubble under tilt angles α between ±5° and ±30°. With increasing α, the magnetic contrast becomes bigger and it reverses for sample tilting to the other direction.

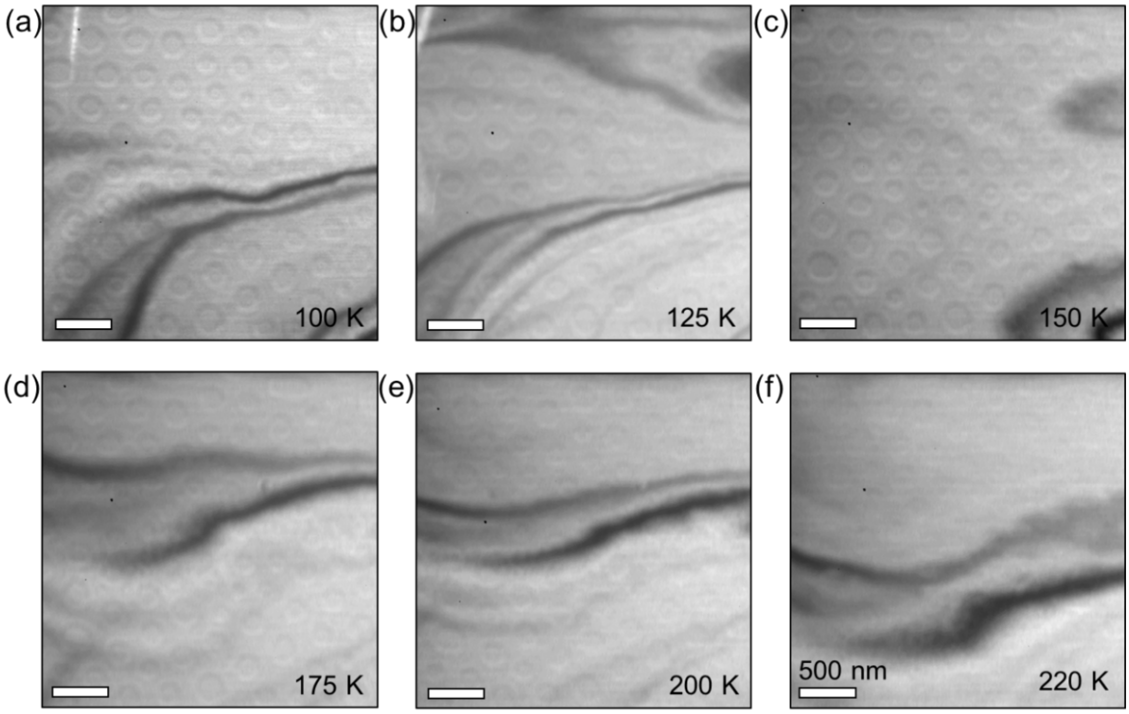

Fig. S15: Néel-type skyrmions in $Cr_{1+\delta}Te_2$ at different temperatures. (a-f) Lorentz transmission electron microscopy (LTEM) micrograph of Néel-type textures acquired at different temperatures in the presence of magnetic field of 160 Oe and at a tilt angle of α= 9°from the [0001] direction. Images were acquired at a defocus of -1.5 mm. The lamella has a thickness of ≈ 75 nm.

Temperature dependent LTEM images were recorded after field cooling under an applied field of 160 Oe applied along the [0001]. Images were collected at constant field at temperature intervals of 25 K within the temperature range of 100-220 K. Warming was carried out at a rate of 2°C/min. The diameter of the Néel-type skyrmions varies only little with temperature (as shown in Fig. 5d) indicating the robust nature of the Néel texture upon thermal excitation. Beyond 175 K the magnetization becomes strongly reduced, therefore the magnetic contrast is considerably diminished as can be seen in Fig. S15d-e. Finally, the magnetic contrast disappears (Fig. S15f) as the temperature exceeds the Curie temperature (≈ 200 K).

References

1. Kuhs, W. Generalized atomic displacements in crystallographic structure analysis. Acta Crystallogr. Sect. A: Found. Crystallogr. **48**, 80 (1992).

2. Hoffmann, M., Ernst, A., Hergert, W., Antonov, V. N., Adeagbo, W. A., Geilhufe, R. M. & Ben Hamed, H. Magnetic and Electronic Properties of Complex Oxides from First-Principles. *Phys.* Status Solidi (b) **257**, 1900671 (2020).

3. Soven, P. Coherent-potential model of substitutional disordered alloys. Phys. Rev. **156**, 809 (1967).

4. Gyorffy, B. Coherent-potential approximation for a nonoverlapping-muffin-tin-potential model of random substitutional alloys. Phys. Rev. B **5**, 2382 (1972).

5. Perdew, J. P. & Wang, Y. Accurate and simple analytic representation of the electron-gas correlation energy. Phys. Rev. B **45**, 13244 (1992).

6. Strange, P., Svane, A., Temmerman, W., Szotek, Z. & Winter, H. Understanding the valency of rare earths from first-principles theory. Nature **399**, 756 (1999).

7. Petit, L., Svane, A., Szotek, Z. & Temmerman, W. First-principles calculations of $PuO_{2\pm x}$. Science **301**, 498 (2003).

8. Turek, I., Kudrnovský, J., Drchal, V. & Bruno, P. Exchange interactions, spin waves, and transition temperatures in itinerant magnets. Philos. Mag. **86**, 1713 (2006).

9. Andersen, O. K. Linear methods in band theory. Phys. Rev. B 12, 3060 (1975).

10. Nemoshkalenko, V., Krasovskii, A., Antonov, V., Antonov, V. N., Fleck, U., Wonn, H. & Ziesche, P. The Relativistic Linear Muffin-Tin Orbital Method Application to Au. Phys. Status Solidi (b) **120**, 283 (1983).

11. Perlov, A. Y., Yaresko, A. N. & Antonov, V. N. PY-LMTO, A Spin-polarized Relativistic Linear Muffin-tin Orbitals Package for Electronic Structure Calculations (1995, unpublished).

12. Chen, J., Wang, L., Zhang, M., Zhou, L., Zhang, R., Jin, L., Wang, X., Qin, H., Qiu, Y. & Mei, J. Evidence for magnetic skyrmions at the interface of ferromagnet/topological-insulator heterostructures. Nano Lett. **19**, 6144 (2019).

13. Zhang, X., Ambhire, S. C., Lu, Q., Niu, W., Cook, J., Jiang, J. S., Hong, D., Alahmed, L., He, L. & Zhang, R. Giant Topological Hall Effect in van der Waals Heterostructures of $CrTe_2/Bi_2Te_3$. ACS nano **15**, 15710 (2021).

14. McVitie, S., Hughes, S., Fallon, K., McFadzean, S., McGrouther, D., Krajnak, M., Legrand, W., Maccariello, D., Collin, S. & Garcia, K. A transmission electron microscope study of Néel skyrmion magnetic textures in multilayer thin film systems with large interfacial chiral interaction. Sci. Rep. **8**, 5703 (2018).
